# Supplementary figures and images for: A New, Discontinuous 2 Phases of Aging Model: Lessons from Drosophila melanogaster
Source: PLoS One. 2015 Nov 3;10(11):e0141920. doi: 10.1371/journal.pone.0141920 (PMC4631373; doi:10.1371/journal.pone.0141920)

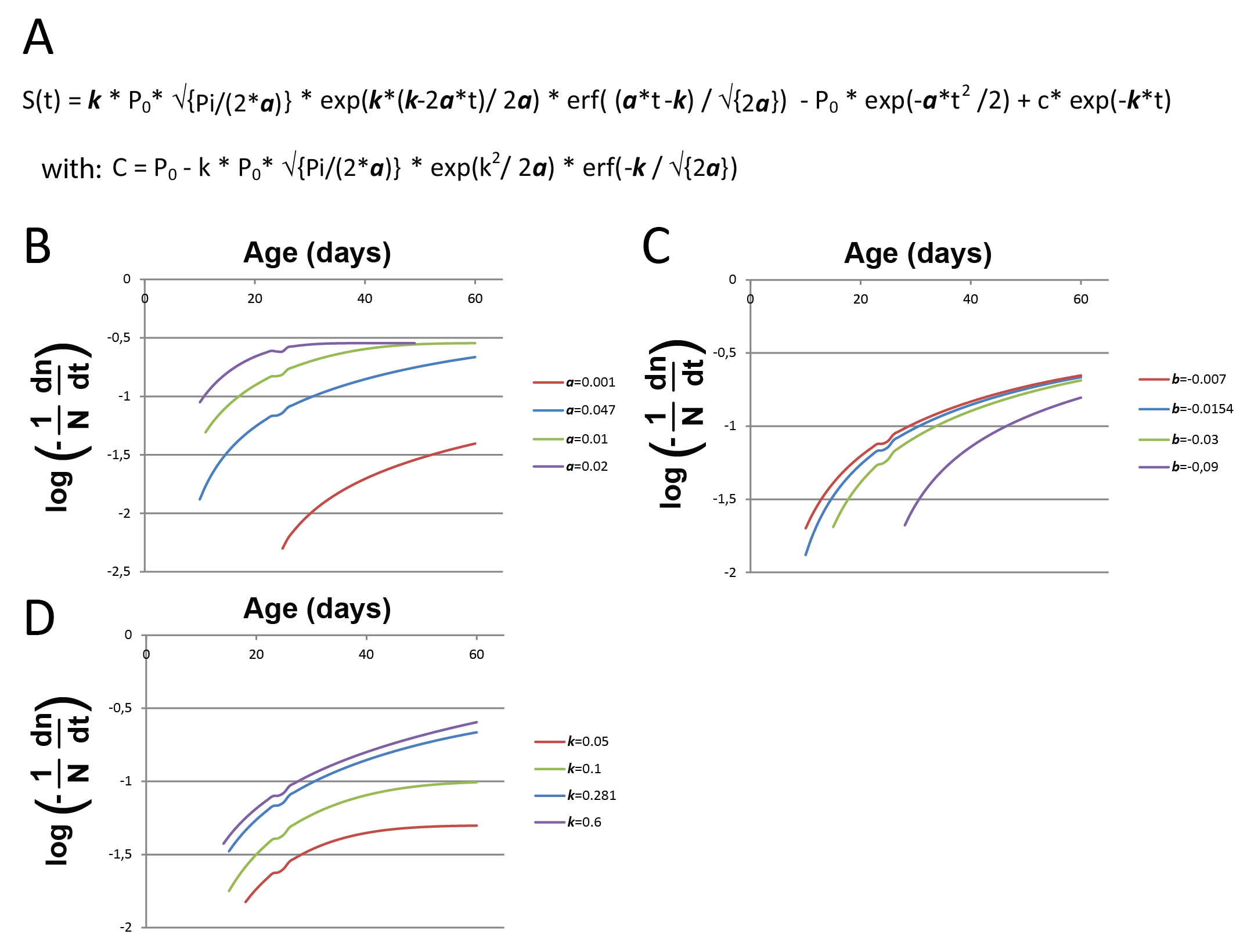

Supplement: S1 Fig — (TIF) [file pone.0141920.s001.tif]

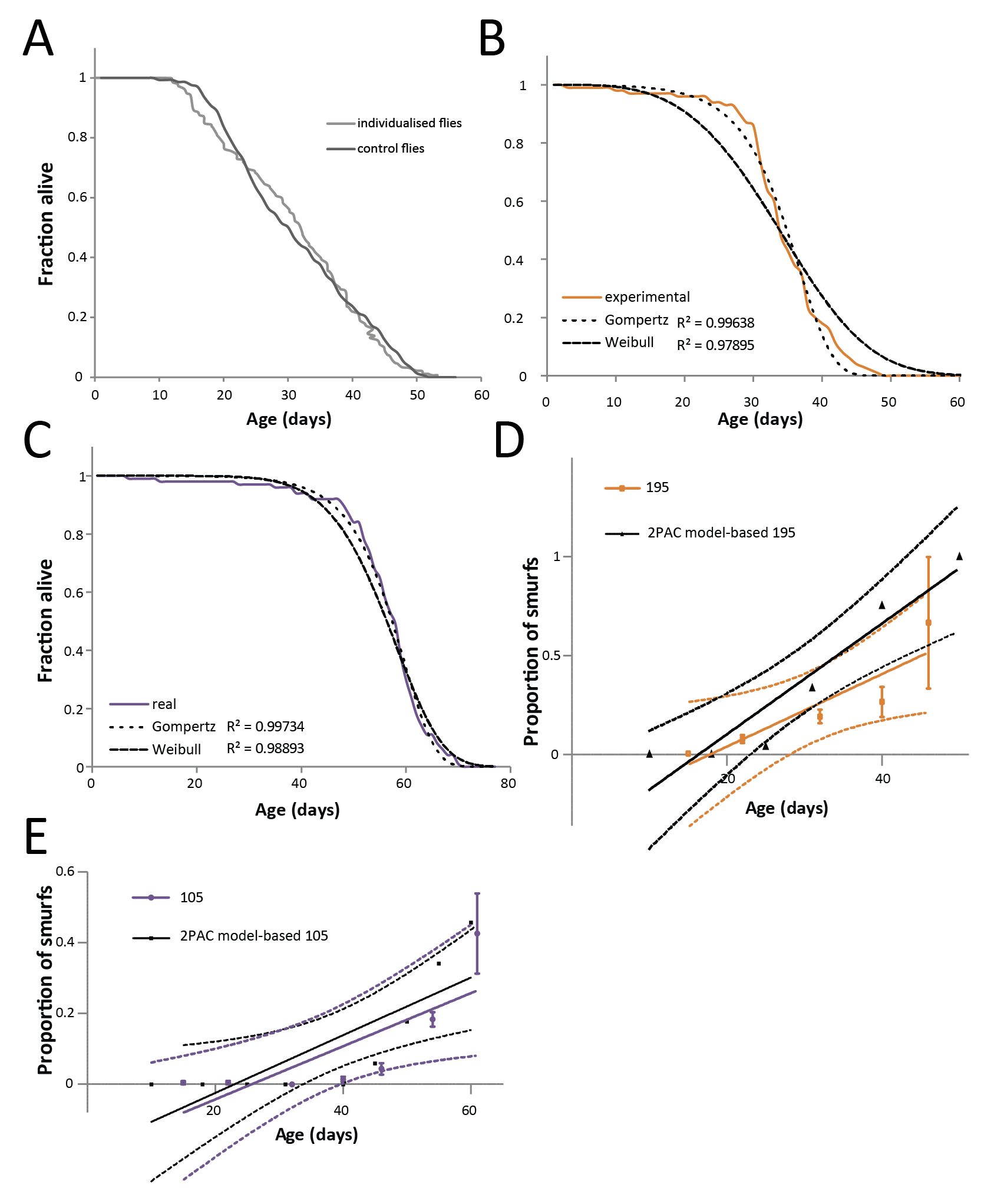

Supplement: S2 Fig — (TIF) [file pone.0141920.s002.tif]

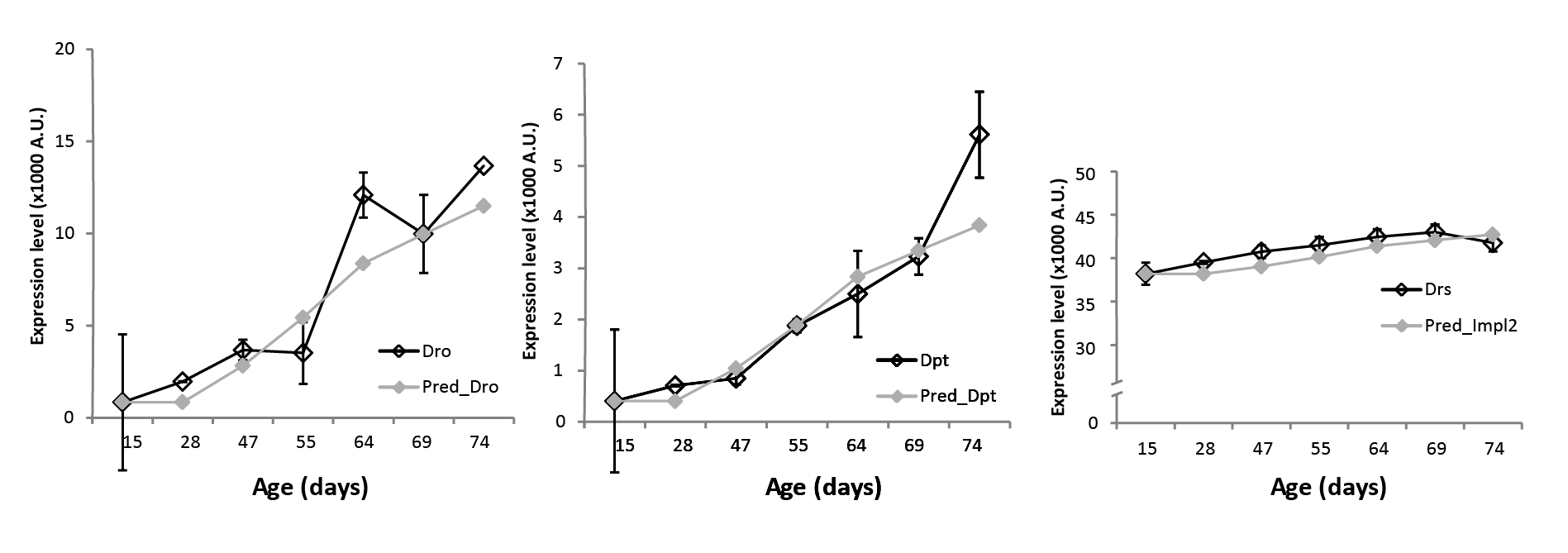

Supplement: S3 Fig — (TIF) [file pone.0141920.s003.tif]
