# Supplementary material for: A New, Discontinuous 2 Phases of Aging Model: Lessons from Drosophila melanogaster
Source: PLoS One. 2015 Nov 3;10(11):e0141920. doi: 10.1371/journal.pone.0141920 (PMC4631373; doi:10.1371/journal.pone.0141920)
Supplement: S1 Text — (DOCX) [file pone.0141920.s004.docx]

**Sup Text1: Detailed analysis of the influence of the different parameters of the 2PAC model on the shape of aging curves**

First, increasing the *daily failure rate* ***a*** results in a dramatic decrease in lifespan of the population, by affecting the length of the initial mortality plateau, the median lifespan T_50_ and the maximum lifespan of the population (Fig 2A). The Smurf Increase Rate (SIR), which is the evolution of the proportion of Smurfs in the population **S_t_/(S_t_+N_t_**), also increases with ***b*** (Fig 2B). Secondly, increasing the *tolerance* parameter **t_0_** increases the length of the initial mortality plateau, the T_50_ and the maximum lifespan of the population (Fig 2C). It also delays the apparition of Smurfs in the population without affecting the rate at which they appear (Fig 2D). We only considered positive value for **t_0_** in these simulations. For a null or a negative value, the proportion of Smurfs would increase from day 0, thus dramatically decreasing or even suppressing the initial mortality plateau. Finally, increasing the *death rate constant* ***k*** does not dramatically affect the length of the initial plateau but decreases both the T_50_ and maximum lifespan of the population. Interestingly, as it affects the turnover of the Smurf population, increasing ***k*** decreases the SIR although it decreases lifespan. This is a case that we have not observed experimentally so far as we only observed a negative correlation between SIR and lifespan.
